# Discovery of the female of *Pyrocoelia prolongata* in Taiwan (Coleoptera, Lampyridae)

**DOI:** 10.3897/zookeys.116.1412

**Published:** 2011-07-07

**Authors:** Ming-Luen Jeng, Michael S. Engel, Ping-Shih Yang

**Affiliations:** 1 Division of Entomology, Natural History Museum, and Department of Ecology & Evolutionary Biology, 1501 Crestline Drive – Suite 140, University of Kansas, Lawrence, Kansas 66045, USA; 2 Entomology Division, Zoology Department, National Museum of Natural Science, 1, Guanqian Rd., Taichung City 40453, Taiwan, ROC; 3 Department of Entomology, National Taiwan University, Taipei City 106, Taiwan, ROC

**Keywords:** *Pyrocoelia prolongata*, female, Taiwan, diurnal, Lampyrinae, Asia

## Abstract

The female of *Pyrocoelia prolongata* Jeng & Lai, a diurnal lampyrid species from Taiwan, is described for the first time. A single individual was found in a small, shady, dry streambed at the edge of a mixed forest at 2700 m elevation. The individual glowed in darkness and would move its abdomen up and down when disturbed and as a deterring behavior. A key to the females of the species of *Pyrocoelia* Gorham in Taiwan is provided. The morphology of photogenic organs of females and the function of bioluminescence of day-active species of *Pyrocoelia* are discussed.

## Introduction

The genus *Pyrocoelia* Gorham is a group of Asian lampyrids including more than 60 known species (McDermott 1966; Jeng et al. 1999b). Males of the genus are alate while, where known, females have vestigial elytra to various degrees and lack hind wings. They can be divided into diurnal and nocturnal groups in which the males possess well-developed photogenic organs in the former but reduced in the latter.

The nomenclature of *Pyrocoelia* was clarified and the species of Taiwan were revised by Jeng et al. (1999b). Several publications provided additional information and images of the females of Taiwanese species (Ho 1997; Chen 1999, 2003; Ho and Chu 2002). Until now, four out of the five species (except *Pyrocoelia prolongata* Jeng & Lai, a species endemic to Taiwan) have confirmed females. Generally it is difficult to find the flightless females in the field, especially for the diurnal species (Ohba 1983). Because of the limited availability and dramatic sexual dimorphism, many of the females were identified to species by rearing larvae to adults. This has not been achieved for *Pyrocoelia prolongata*. Fortunately a female of the species was discovered in the field during a collecting trip to high mountains in southern Taiwan in 2004. Herein we describe this individual and its remarkable bionomics, and provide a key to the females of Taiwan’s species of the genus.

## Material and methods

The only female specimen available for study was collected by the late Japanese coleopterist Dr. M. Satô while digging in a dry streambed for ground beetles at high elevations in southern Taiwan. The female was kept in a transparent plastic cup which was spread with wet tissue paper and moss to maintain suitable humidity and for observation.

Measurements were made by outlining the target structures under a Nikon SMZ-10 microscope with a camera lucida. Body length (BL) was measured as the distance between pronotal and abdominal apices when the body was fully stretched, and body width (BW) as the greatest distance across the abdomen. The abbreviations PL and PW denote pronotal length and width, respectively; while T#, P#, and S# refer to the tergite, pleurite, and sternite of the #th abdominal segment (true segmentation), respectively.

## Systematics

### Pyrocoelia prolongata

Jeng and Lai

http://species-id.net/wiki/Pyrocoelia_prolongata

Pyrocoelia prolongata Jeng & Lai, *in*Jeng et al. 1999b: 358.

#### Diagnosis.

 The female is characterized by the bell-shaped pronotum which resembles that of the male and is one of the diagnostic characters of the species (Jeng et al. 1999b). No other congeneric species from Taiwan share a similar pronotal morphology and coloration in the female. The temporal and spatial distribution also fits that of *Pyrocoelia prolongata*.

#### Description.

**Figure 1.**
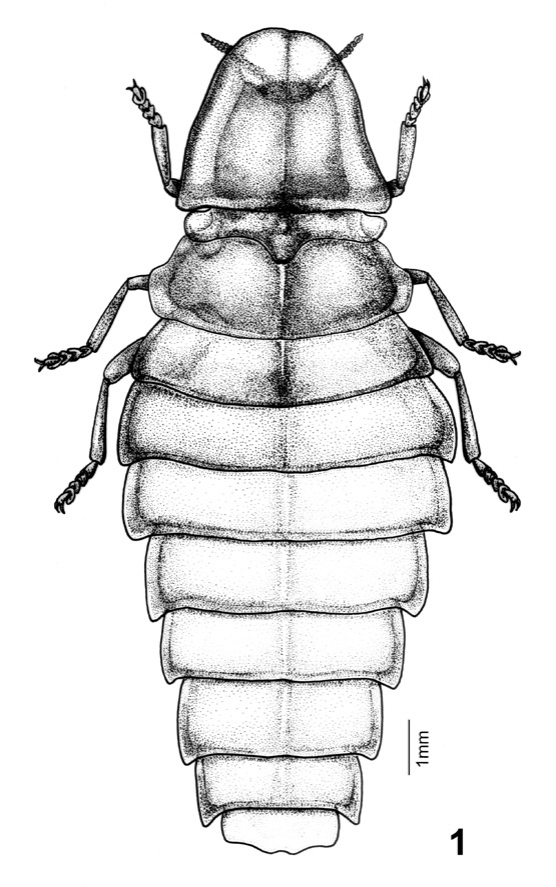
Female dorsal habitus of *Pyrocoelia prolongata* Jeng and Lai.

**Figures 2–6.**
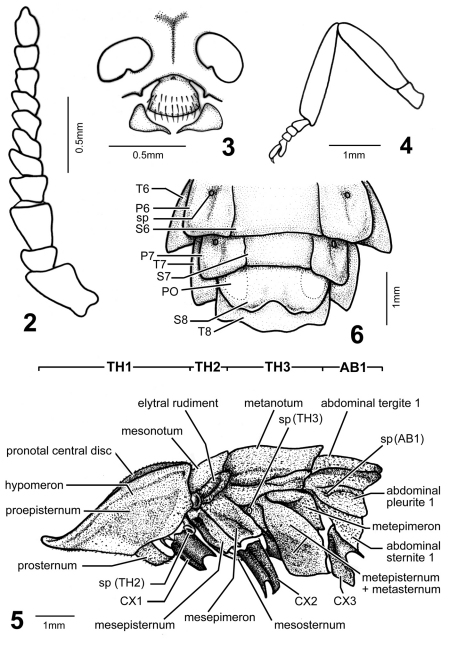
*Pyrocoelia prolongata* Jeng and Lai, female **2** Antenna **3** Partial head in frontal aspect (frons, antennal sockets, clypeus-labrum, and mandibles), antennae removed **4** Hind leg (metatrochanter–metatarsus) **5** Thoracic segments (TH1–3), coxae (CX1–3), and first abdominal segment (AB1), lateral aspect; sp (#) = spiracle on #th segment **6** Abdominal segments 6–8, ventral aspect, T#, P#, and S# = abdominal tergite, pleurite, and sternite of #th segment; PO = photogenic organs.

**Figure 7.**
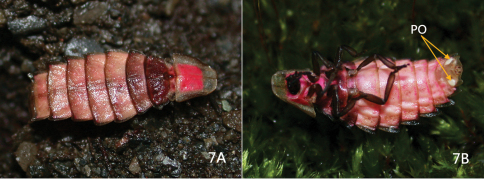
Photos of female of *Pyrocoelia prolongata* Jeng & Lai **A** Dorsal aspect **B** Ventrolateral oblique aspect. Note the pink and bronze luster on the dorsum as well as the distinct photogenic organs (PO) on the venter.

*Female* (Figs 1–7): BL: 15.6 mm; BW: 4.6 mm. Body form elliptical and depressed. Head, antennae, and legs dark brown to black; pronotum translucent gray, with margins and central carina light brown and central disc pink; mesonotum pink, central disc and mesoscutellum dark brown, translucent gray at sides (elytral rudiments); metanotum bronze; abdominal T1 lighter than metanotum and mixed of pink; T2–7 pinkish white, bronze on posterior margins and central line, and translucent gray at lateral margins (Fig. 7A); T8 translucent gray; ventral side pink, brightly so on thorax and pale on abdomen; S8 with a pair of milky white photogenic organs; S8–9 translucent yellowish brown. Head spherical, about 1/3 as broad as pronotum; vertex concave along central line; frons about as broad as clypeus-labrum; antennal sockets widely separated from each other; antenna (Fig. 2) 11-segmented, weakly serrate and rod-like; antennomere 1 largest and 3 slightly smaller than it; clypeus-labrum (Fig. 3) not fused with frons, shell shape; mandibles partially sclerotized, thick in basal 2/3 and slender and somewhat curved at apex; maxillary palpus 4-segmented; labial palpus 3-segmented. Pronotum bell-shape, strongly convex on central disc and weakly so in windows, reflexed at margins; windows on apical 1/3 and divided by central carina. PL/PW = 0.85. Mesonotum as broad as central disc of pronotum, with elytral rudiments abbreviated and round at sides, not extending beyond pronotum; mesoscutellum transverse, broader than long by 2 times, with apex slightly notched. Metanotum broader than pronotum, transversely elliptical, with a concave central line. Metepisternum fused with metasternum (Fig. 5). Legs (Fig. 4) moderate in width and length, 5th tarsomere slightly longer than preceding tarsomere. Abdomen broadest at T2, T1 about as broad as T3 and diminishing in width accordingly toward apex; T1–8 each with short but clear hind angles; T1–7 with a paler central line; T8 (Fig. 6) transverse and subquadrate, sinuate at sides and central apex. P1–7 folded on inner margins, each bearing a spiracle at center. S8 deeply notched centrally, with a pair of photogenic organs at sides (= PO, Fig. 6, Fig. 7B).

#### Material examined.

 1♀, S. Taiwan: Kaohsiung County (now Kaohsiung City), Yako logging trail (abandoned), 120º57’E, 23º16’N, 2700m above sea level, 8.VII.2004 around midday, M. Satô leg.; specimen deposited in the collection of the senior author, National Museum of Natural Science, Taiwan.

#### Sexual dimorphism.

Females differ from males by (generic level differences) **1**, having shorter and less serrate antennae, much smaller compound eyes, and smaller palpi; **2**, the mesoscutellum as broad as long; **3**, the highly abbreviated elytra and absence of hind wings; **4**, the fusion of metepisternum and metasternum; **5**, having an intact abdominal S1; **6**, having the hind angles of abdominal T1–7 not very acute; **7**, S8 being deeply emarginate (cf., Jeng et al. 1999b); **8**, (remainder are species-level differences) the bronze on the metanotum and abdominal T1; and **9**, the narrower dark margins on the pronotum. In contrast to known females of *Pyrocoelia*, the female of *Pyrocoelia prolongata* is smaller than its respective males. We are not sure if this is simply individual variation or universal to the species.

#### Habitat and phenology.

**Figure 8.**
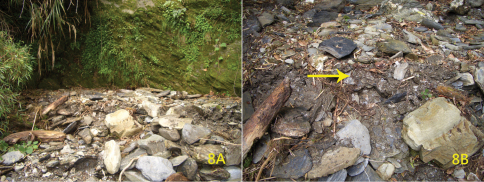
Habitat of *Pyrocoelia prolongata* Jeng & Lai female **A** General locality in Yako logging trail, a dry streambed at 2700 m above sea level, southern Taiwan **B** Close-up, indicating where the female was discovered (arrow).

 The female was found in a small, shady, and damp dry streambed at the edge of a mixed forest dominant with Taiwan Red False Cypress [*Chamaecyparis formosensis* Matsumura (Cupresaceae)] (Fig. 8A). It stayed on the substrate mixed of gravel and sand under a slate stone (Fig. 8B). An Alishan salamander (*Hynobius arisanensis* Maki) was also found in the microhabitat. Only a few species of Lampyrinae such as *Diaphanes nubilus* Jeng & Lai, *Pyrocoelia formosana* Olivier, and *Pyrocoelia prolongata* have ever been recorded from localities at such high elevations in Taiwan (Jeng et al. 1999a, some of the species denoted as “sp.” at that time). No living male of *Pyrocoelia prolongata* was found in or around the locality during the 3-day duration of our collecting trip, except for an already dead individual. This suggests that the mating season might have come to an end. At that time, a population of the species in central Taiwan (Anmashan, Taichung County, alt. 2300m) was in a peak season of activity (C.F. Lee, pers. comm.).

#### Behavioral remarks.

 A male of *Pyrocoelia formosana* collected from the same locality was put together with the female in a transparent plastic container to observe their interaction. At first the male did not show interest in the female. Later it attempted to copulate with the female but failed due to the female’s resistance. They eventually copulated after one day of captivity. The male perished soon after copulation, while the female survived the following week and laid three or four eggs before she died. All eggs failed to hatch and decomposed. The female glowed in darkness via a pair of photogenic organs on abdominal sternite 8. When wagging a finger around the female’s head in a dusky environment, the female responded by powerfully raising her abdomen vertically to the body axis then laid down. It was found to be a one-to-one response after several repetitions. Apparently the female can detect the nearby moving subject and the action could be an intimidation to predators.

##### Key to the females of Pyrocoelia species of Taiwan

**Table:** 

1	Elytral rudiments clearly extending beyond pronotum in width	2
–	Elytral rudiments short, not or slightly extending beyond pronotum in width	4
2	Elytral rudiments broad, lobe-like, extending posterolaterally and covering part of metanotum	3
–	Elytral rudiments narrow, sword-like, extending laterally and not covering any part of metanotum	*Pyrocoelia sanguiniventer* Olivier
3	Elytral rudiments longer than broad by 1.5 times; mesoscutellum about as broad as long; pronotal windows ambiguous	*Pyrocoelia analis* (F.)
–	Elytral rudiments longer than broad by 3 times; mesoscutellum transverse; pronotal windows clear	*Pyrocoelia praetexta* Olivier
4	Abdomen without morphologically recognizable photogenic organs; metanotum milky white; pronotum semicircular, translucent gray in explanate area	*Pyrocoelia formosana* Olivier
–	Abdomen with a pair of photogenic organs on sternite 8; metanotum bronze; pronotum more-or-less projecting forward, dark in margins and central carina between windows	*Pyrocoelia prolongata* Jeng & Lai

## Discussion

The photogenic organs of females *Pyrocoelia* vary in number and position. Among the known taxa from Japan and Taiwan, *Pyrocoelia analis*, *Pyrocoelia praetexta*, *Pyrocoelia rufa* Olivier, *Pyrocoelia miyako* Nakane, and *Pyrocoelia atripennis* Lewis each have two pairs of morphologically recognizable lanterns on sternites 6 and 7 (Ohba 1983, 2004; Ho 1997, 2003; Chen 1999, 2003); *Pyrocoelia prolongata* has a significant pair on sternite 8; while *Pyrocoelia formosana*, *Pyrocoelia sanguiniventer*, *Pyrocoelia discicollis* Kiesenwetter, *Pyrocoelia matsumurai* Nakane, and *Pyrocoelia abdominalis* Nakane do not have visible lanterns if not glowing (Ohba 1993). However, females of *Pyrocoelia praetexta* are able to emit six spots of light on sternites 6–8 and at least some species in the last group are confirmed to glow weakly in two spots on sternite 8 (Ho and Chu 2002; Chen 2003; Ohba 2004). It is therefore somewhat risky to interpret glowing behavior based solely on the external morphology of dead, and sometimes dried, specimens.

Both sexes of *Pyrocoelia prolongata* can emit green light from a pair of lanterns on sternite 8. The males are active during the daytime to locate mates by raising their heads and elevating the antennae in a V-shaped position, used to detect female pheromones, as observed for many other diurnal species (Ohba 1983, 1997a, 1997b, 2004). Occasionally they can be found at night owing to their bioluminescence. (Ohba (1983, 2004) classified the diurnal species of *Pyrocoelia* from Japan into the CR system in which males find mates relying mainly on chemical signals and with the aid of relatively weak luminescent signals from mates over a short distance. However, the females of the diurnal species of *Pyrocoelia*, though able to glow, are extremely rarely seen in the field at night (Ohba 1983). The function of the female bioluminescence is thus questionable. Lall and Lloyd (1989) studied a day-active species, *Lucidota luteicollis* LeConte, and found that the electroretinographic sensitivity of the male (lamda-maximum= 530 nm) did not match its emission spectrum (lamda-maximum= 562 nm). These authors concluded that the maledoes not rely on light signals to find a mate, and suggested that the flightless female might be the light receiver. The female could use the male bioluminescence to recognize a mate in the shady, dusky microhabitat in which such long-wavelength light can be transmitted more so than short wavelengths, and thus be detectable by the female. The bioluminescent spectrum and electroretinographic sensitivity of *Pyrocoelia* are scarcely studied except for a few cases (Eguchi et al. 1984; Ohmiya et al. 1996). The night-active *Pyrocoelia miyako* Nakane emits green light with a lambda-maximum at 550 nm by males (Ohmiya et al. 1996). The electroretinograph of *Pyrocoelia atripennis* Nakane, a day-active species from Japan, shows a broad green sensitivity with the lambda-maximum around 540 nm in the male (Eguchi et al. 1984). There is not yet any study on the bioluminescence and visual sensitivity in both sexes of a diurnal species of *Pyrocoelia*. Whether the bioluminescence is truly functional or merely an evolutionary residual remains open for future study.

## Supplementary Material

XML Treatment for Pyrocoelia prolongata
